# Prevalence and Severity of Periodontal Disease in Diabetic Patients in South Jordan: A Cross-Sectional Study

**DOI:** 10.7759/cureus.66203

**Published:** 2024-08-05

**Authors:** Marwan Showayter, Mohammad Aljariri, Ahmed Al dalalah, Hossam Al-Fuqaha, Ahmad AlKhatib, Abeer Mohammad, Saif Aburumman

**Affiliations:** 1 Periodontics, Royal Medical Services, Amman, JOR; 2 Conservative Dentistry and Endodontics, Royal Medical Services, Amman, JOR; 3 Prosthodontics, Royal Medical Services, Amman, JOR; 4 Nursing, Royal Medical Services, Amman, JOR; 5 Medicine, University of Jordan, Amman, JOR

**Keywords:** community periodontal index, papillary bleeding index, gingival index, plaque index, periodontal disease, duration of diabetes mellitus

## Abstract

Introduction: Poorly managed diabetes mellitus can elevate oral glucose levels, fostering gum disease. Conversely, untreated periodontal disease may worsen diabetes control. This study aims to assess the prevalence of periodontal disease and its association with diabetes characteristics in South Jordan.

Methods: This cross-sectional study enrolled 249 type 2 diabetic patients from Prince Hashim Bin Abdullah II Clinic in Aqaba, Jordan. Demographics, clinical history, and periodontal indices were recorded, with glycemic control measured via HbA1c. Statistical analyses utilized SPSS.

Results: Predominantly female (58%) and married (90%) participants had a mean age of 49.0 years, with uncontrolled diabetes prevalent in 86% (mean HbA1c: 9.16). Dyslipidemia (73%), hypertension (49%), and diabetic neuropathy (21%) were common. Periodontal indices indicated moderate to high scores, reflecting significant plaque accumulation (plaque index score (PIS) = 3: 20%), severe gingival inflammation (gingival index score (GIS) = 3: 22%), and notable bleeding upon probing (papillary bleeding index score (PBIS) = 3-4: 22%). Moreover, a considerable percentage exhibited advanced periodontal disease (community periodontal index score (CPIS) = 3-4: 19%).

Conclusion: A high prevalence of periodontal disease among diabetic patients in South Jordan underscores the need for integrated diabetes and periodontal care strategies. These findings emphasize the interplay between diabetes control and periodontal health, warranting further investigation into effective intervention strategies.

## Introduction

Diabetes mellitus (DM) is a metabolic disorder characterized by abnormally high levels of blood glucose, encompassing various categories, including type 1 diabetes, type 2 diabetes, maturity-onset diabetes of the young (MODY), gestational diabetes, neonatal diabetes, and secondary forms resulting from endocrinopathies, steroid use, among others. The primary subtypes of DM are type 1 diabetes mellitus (T1DM) and type 2 diabetes mellitus (T2DM), typically arising from impaired insulin secretion (T1DM) and/or function (T2DM) [1,2].

With its continuously rising global occurrence, diabetes has become a paramount and formidable health concern for the current global population. The rise in diabetes prevalence in numerous regions worldwide correlates with swift economic growth, urbanization, and the adoption of contemporary lifestyle behaviors [3]. This has been more rapid in low- and middle-income nations compared to high-income ones. This escalation may stem from a heightened incidence of diabetes, improved survival rates, population aging, or a blend of these elements. In the USA, diabetes incidence nearly doubled from 1970 to 2000. Similarly, in Jordan, diabetes prevalence exhibited an increase from 1994 to 2004 [4,5].

Ajlouni et al. showed that the prevalence rate surged rapidly from 1994 to 2009 but then decelerated, and a spike was notably higher among men than women. A significant portion of all diabetes cases in the four surveys were previously diagnosed, with the highest percentage observed in the 2017 survey. This trend suggests that the national diabetes strategy may be yielding beneficial outcomes [6].

Periodontal disease has been associated with various systemic diseases and conditions, such as cardiovascular issues, kidney problems, autoimmune disorders, respiratory ailments, hormonal imbalances, neurodegenerative diseases, and cancer [7]. Numerous studies indicate that individuals with poorly managed diabetes or those struggling to control their blood glucose levels face a two to three times greater risk of developing periodontitis, with the level of glycemic control serving as the primary determinant of risk [8].

Periodontal disease can exert a notable influence on the metabolic condition of individuals with diabetes. Recent literature suggests that treating periodontal disease could potentially enhance glucose control. The elevated levels of pro-inflammatory substances found in the gums of individuals with poorly managed diabetes indicate the presence of a biological pathway that could exacerbate periodontitis [9,10].

Understanding the link between oral inflammation in periodontal disease and systemic inflammation is crucial for comprehending the potential long-term negative impacts of periodontal inflammation on the systemic function of various organs. This exploration may help reveal the degree to which oral diseases contribute to the risk of developing non-oral conditions [11]. Therefore, in this cross-sectional single-center study, we aim to investigate the association between the severity of periodontitis and the course of DM in the Jordanian population.

## Materials and methods

Study design

We carried out a cross-sectional observational study at Prince Hashim Bin Abdullah II Clinic in Aqaba, south of Jordan, between February 2024 and May 2024, including a total of 249 patients with DM.

The study included both male and female patients diagnosed with type I or II DM at any age. However, the DM patients who had complications, systemic disease other than DM, and underwent periodontal treatment during the past six months were excluded from this study. Lactating or pregnant women were excluded. Those who did not agree to participate in this study were excluded.

Two calibrated examiners performed a full mouth periodontal examination and periodontal charts for all the participants to determine the periodontal status and periodontitis stage using the World Health Organization (WHO) basic periodontal examination (BPE) probe with a “ball end” of 0.5 mm in diameter and a black band from 3.5 to 5.5 mm.

Data collection

Retrospective data collection involved accessing hospital records to gather demographic variables such as age, gender, marital status, income, educational level, and smoking history. Clinical data encompassed variables including body mass index (BMI), duration of DM, DM control, DM medications (oral antihyperglycemic or insulin), glycated hemoglobin (HbA1c) levels, hypertension history, dyslipidemia, cardiovascular diseases, and DM complications. Dental characteristics were evaluated using periodontal indices such as the plaque index, gingival index, papillary bleeding index, and community periodontal index. Qualitative indices were employed to assess the inflammatory diseases affecting the gingiva and periodontium, along with their associated symptoms and causative agents, such as microbial plaque or biofilm. Table 1 outlines the indexing systems employed to assess the periodontal status of the included patients.

**Table 1:** The periodontal indices used in the study

Plaque index	
0	No plaque in the gingival area
1	A film of plaque adhering to the free gingival margin and adjacent area of the tooth; may be recognized only by running a probe across the tooth surface
2	Moderate accumulation of soft deposits within the gingival pocket and on the gingival margin and/or adjacent tooth surface; can be seen by the naked eye
3	Abundance of soft material within the gingival pocket and/or on the gingival margin and adjacent tooth surface
Papillary bleeding index	
0	No bleeding on probing
1	Single ecchymosis of the gingiva on probing
2	Multiple ecchymoses or minor single spot extravasation from the gingiva on probing
3	Bleeding into the pocket immediately after probe insertion
4	Intensive extra pocket bleeding on probing
Gingival index	
0	Normal gingiva
1	Mild inflammation, slight change in color, slight edema, no bleeding on palpation
2	Moderate inflammation, redness, edema, glazing, bleeding on palpation
3	Severe inflammation, marked redness, edema, ulceration, tendency to spontaneous bleeding
Community periodontal index	
0	Healthy gingiva
1	Bleeding observed, directly or by using a mouth mirror, after probing
2	Calculus detected during probing, but all the black bands on the probe were visible
3	Pocket 4–5 mm (gingival margin within the black band on the probe)
4	Pocket 6 mm or more (black band on the probe not visible)
X	Excluded sextant (less than two teeth present)

Statistical analysis

For continuous variables, the mean ± standard deviation (SD) was reported if the data exhibited a normal distribution, as confirmed by the Shapiro-Wilk test. In cases where normality assumptions were violated, the median (Q1, Q3) was used instead. Categorical variables were summarized using frequencies (percentages, %). The association between demographic, clinical, and laboratory variables with study groups was evaluated using the Wilcoxon (Mann-Whitney U) test for continuous variables, while the chi-squared (X^2^) and Fisher's exact tests were applied for categorical variables, especially when the category count was less than 5. Statistical significance was considered at a p-value of <0.05. All statistical analyses were conducted using the R software package (version 4.3.1).

## Results

Baseline characteristics

A total of 249 diabetic patients were included with a mean age of 49.0 (8.0) years. The majority (58%) of patients were females, and 224 (90%) were married. The educational level was lower than secondary in 100 (40%) patients, with 130 (52%) patients having a monthly income between 500 and 1000 Jordanian Dinars (JODs). The mean BMI level was 31.4 (5.2), with a mean DM duration of 8.2 (5.1) months. Diabetes was uncontrolled in 214 (86%) patients, 165 (66%) patients were on oral hypoglycemic agent (OHA) alone, and 77 (31%) were on OHA plus insulin. The mean HbA1c level was 9.16 (2.01); 181 (73%) had dyslipidemia, 122 (49%) had hypertension, and 46 (18%) had cardiovascular diseases. Diabetic neuropathy was seen in 53 (21%) patients, retinopathy in 22 (8.8%) patients, and nephropathy in 12 (4.8%) patients. Table 2 shows the baseline demographic and clinical characteristics of included patients.

**Table 2:** Demographic and clinical characteristics of included patients based on the plaque score index and the gingival index score ^ 1 ^Mean (SD); n (%). ^2^ Kruskal-Wallis rank sum test; Pearson’s Chi-squared test; and Fisher’s exact test. JODs: Jordanian Dinars.

Characteristics	Plaque score index				p-value^2^	Gingival index score				p-value^2^	Overall
	0 N = 19^1^	1 N = 30^1^	2 N = 149^1^	3 N = 51^1^		0 N = 21^1^	1 N = 31^1^	2 N = 142^1^	3 N = 55^1^		N = 249^1^
Age	47 (7)	42 (11)	48 (7)	54 (5)	<0.001	43 (8)	41 (9)	49 (7)	54 (3)	<0.001	49 (8)
Gender					0.044					0.061	
Female	12 (63%)	16 (53%)	78 (52%)	38 (75%)		11 (52%)	13 (42%)	81 (57%)	39 (71%)		144 (58%)
Male	7 (37%)	14 (47%)	71 (48%)	13 (25%)		10 (48%)	18 (58%)	61 (43%)	16 (29%)		105 (42%)
Marital status					0.4					0.8	
Divorced or widowed	0 (0%)	1 (3.3%)	5 (3.4%)	5 (9.8%)		0 (0%)	1 (3.2%)	7 (4.9%)	3 (5.5%)		11 (4.4%)
Married	19 (100%)	26 (87%)	135 (91%)	44 (86%)		20 (95%)	28 (90%)	125 (88%)	51 (93%)		224 (90%)
Single	0 (0%)	3 (10%)	9 (6.0%)	2 (3.9%)		1 (4.8%)	2 (6.5%)	10 (7.0%)	1 (1.8%)		14 (5.6%)
Education					0.11					0.3	
	7 (37%)	9 (30%)	54 (36%)	30 (59%)		7 (33%)	11 (35%)	53 (37%)	29 (53%)		100 (40%)
>Secondary	5 (26%)	6 (20%)	36 (24%)	7 (14%)		7 (33%)	8 (26%)	28 (20%)	11 (20%)		54 (22%)
Secondary	7 (37%)	15 (50%)	59 (40%)	14 (27%)		7 (33%)	12 (39%)	61 (43%)	15 (27%)		95 (38%)
Income (JODs)					0.5					0.8	
500	11 (58%)	17 (57%)	66 (44%)	25 (49%)		12 (57%)	16 (52%)	66 (46%)	25 (45%)		119 (48%)
500-1000	8 (42%)	13 (43%)	83 (56%)	26 (51%)		9 (43%)	15 (48%)	76 (54%)	30 (55%)		130 (52%)
BMI	29.0 (4.3)	30.2 (5.4)	31.8 (5.4)	32.0 (4.8)	0.072	28.3 (4.0)	31.4 (6.6)	32.0 (5.0)	31.1 (5.2)	0.016	31.4 (5.2)
DM duration	6.2 (3.5)	4.5 (4.1)	7.5 (4.1)	13.3 (5.0)	<0.001	4.5 (3.5)	4.1 (3.0)	8.4 (5.1)	11.5 (3.9)	<0.001	8.2 (5.1)
Glycemic control					<0.001					<0.001	
Controlled	15 (79%)	11 (37%)	5 (3.4%)	4 (7.8%)		11 (52%)	10 (32%)	10 (7.0%)	4 (7.3%)		35 (14%)
Uncontrolled	4 (21%)	19 (63%)	144 (97%)	47 (92%)		10 (48%)	21 (68%)	132 (93%)	51 (93%)		214 (86%)
DM medications					0.3					0.066	
Insulin	0 (0%)	1 (3.3%)	4 (2.7%)	2 (3.9%)		0 (0%)	1 (3.2%)	3 (2.1%)	3 (5.5%)		7 (2.8%)
OHA	17 (89%)	22 (73%)	96 (64%)	30 (59%)		19 (90%)	24 (77%)	89 (63%)	33 (60%)		165 (66%)
OHA + Insulin	2 (11%)	7 (23%)	49 (33%)	19 (37%)		2 (9.5%)	6 (19%)	50 (35%)	19 (35%)		77 (31%)
Smoking status					0.073					0.023	
Former smoker	4 (21%)	2 (6.7%)	11 (7.4%)	5 (9.8%)		3 (14%)	2 (6.5%)	14 (9.9%)	3 (5.5%)		22 (8.8%)
Non-smoker	8 (42%)	13 (43%)	75 (50%)	34 (67%)		5 (24%)	15 (48%)	72 (51%)	38 (69%)		130 (52%)
Smoker	7 (37%)	15 (50%)	63 (42%)	12 (24%)		13 (62%)	14 (45%)	56 (39%)	14 (25%)		97 (39%)
HbA1c level	6.53 (1.53)	8.41 (1.88)	9.18 (1.42)	10.53 (2.50)	<0.001	7.50 (1.54)	8.21 (1.97)	9.43 (1.73)	9.63 (2.37)	<0.001	9.16 (2.01)
Comorbidities											
Dyslipidemia	11 (58%)	7 (23%)	117 (79%)	46 (90%)	<0.001	6 (29%)	25 (81%)	110 (77%)	40 (73%)	<0.001	181 (73%)
Hypertension	4 (21%)	6 (20%)	77 (52%)	35 (69%)	<0.001	2 (9.5%)	11 (35%)	77 (54%)	32 (58%)	<0.001	122 (49%)
CVD	3 (16%)	1 (3.3%)	26 (17%)	16 (31%)	0.013	0 (0%)	5 (16%)	30 (21%)	11 (20%)	0.088	46 (18%)
Neuropathy	3 (16%)	2 (6.7%)	28 (19%)	20 (39%)	0.003	1 (4.8%)	5 (16%)	33 (23%)	14 (25%)	0.2	53 (21%)
Retinopathy	0 (0%)	1 (3.3%)	14 (9.4%)	7 (14%)	0.3	0 (0%)	2 (6.5%)	11 (7.7%)	9 (16%)	0.13	22 (8.8%)
Nephropathy	0 (0%)	1 (3.3%)	9 (6.0%)	2 (3.9%)	0.9	0 (0%)	2 (6.5%)	5 (3.5%)	5 (9.1%)	0.3	12 (4.8%)

Periodontal indices

**Figure 1:**
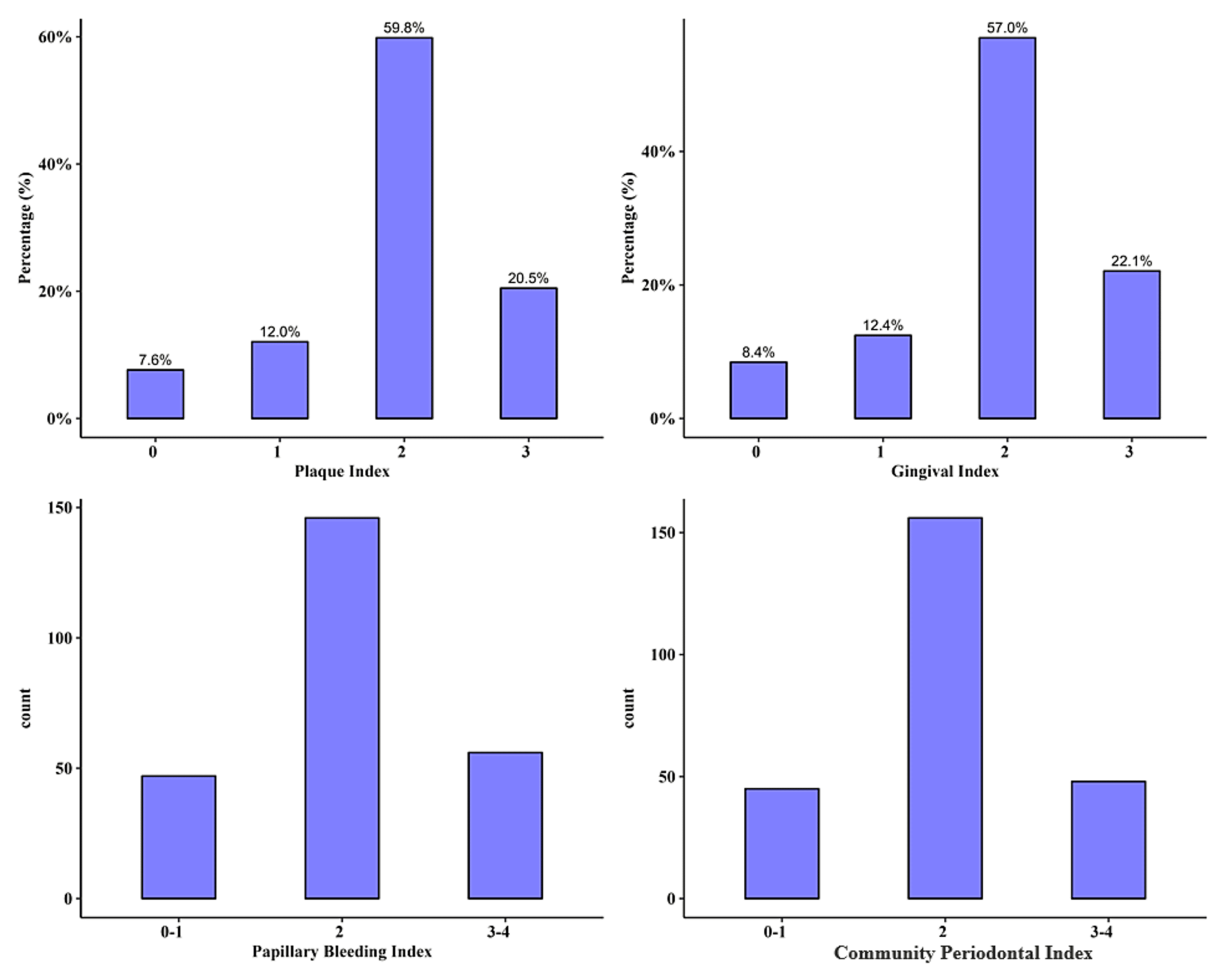
Distribution of periodontal indices in the included patients (A) Plaque index, (B) gingival index, (C) papillary bleeding index, and (D) community periodontal index.

The plaque index score (PIS) was on the intermediate level (PIS = 2) in 149 (60%) patients and high (PIS = 3) in 51 (20%) patients. For the gingival index score (GIS), 55 (22%) had a GIS score of 3, and 142 (59%) had a GIS of 2. The majority (59%) of patients had a papillary bleeding index score (PBIS) of 2, while 56 (22%) of patients had a PBIS between 3 and 4. Lastly, the community periodontal index score (CPIS) was 2 in 156 (63%) patients, and 3-4 in 48 (19%) patients. Figure 1 shows the distribution of the periodontal indices for our study.

Association between DM and periodontal indices

When comparing the association of DM characteristics with the PIS, patients with higher PIS scores (PIS = 3) were associated with higher age (p-value < .001), and 75% of patients with a PIS of 3 were females (p-value = .044). Longer DM duration was associated with higher PIS, and 47 (92%) patients with PIS of 3 had uncontrolled DM (p-values < .001). Hb1Ac was significantly higher in patients with PIS of 3; 46 (90%) patients with high PIS had dyslipidemia, 35 (69%) had hypertension, 16 (31%) had cardiovascular diseases, and 20 (39%) had diabetic neuropathy (Table 2).

For the GIS, patients with higher GIS scores (GIS = 3) were also associated with higher age (p-value < .001), and a significantly higher BMI was seen in patients with GIS of 2 (Table 2). Longer DM duration was associated with higher GIS, and 51 (93%) patients with GIS of 3 had uncontrolled DM (p-values < .001). Hb1Ac was significantly higher in patients with GIS of 3, 110 (77%) patients with a GIS of 2 had dyslipidemia, and 77 (54%) had hypertension.

A higher PBIS score (PBIS = 3-4) was associated with higher age (p-value = .004). Longer DM duration was associated with higher PBIS, and 52 (93%) patients with PBIS of 3-4 had uncontrolled DM (p-values < .05). Hb1Ac was significantly higher in patients with PBIS of 3-4, 42 (75%) patients with a PBIS of 3-4 had dyslipidemia, and 10 (18%) had diabetic retinopathy (Table 3).

**Table 3:** Demographic and clinical characteristics based on the papillary bleeding index score and the periodontal index score ^1 ^Mean (SD); n (%). ^2 ^Kruskal-Wallis rank sum test; Pearson’s Chi-squared test; and Fisher’s exact test. JODs: Jordanian Dinars; OHA: Oral hypoglycemic agents; CVD: Cardiovascular disease.

Characteristics	Papillary bleeding index				Periodontal index score			p-value^2^	Overall
	0-1 N = 47^1^	2 N = 146^1^	3-4 N = 56^1^	p-value^2^	0-1 N = 45^1^	2 N = 156^1^	3_4 N = 48^1^	p-value^2^	N = 249^1^
Age	46 (8)	49 (8)	51 (6)	0.004	46 (9)	47 (8)	55 (5)	<0.001	49 (8)
Gender				0.2				0.5	
Female	22 (47%)	88 (60%)	34 (61%)		24 (53%)	89 (57%)	31 (65%)		144 (58%)
Male	25 (53%)	58 (40%)	22 (39%)		21 (47%)	67 (43%)	17 (35%)		105 (42%)
Marital status				0.6				>0.9	
Divorced or widowed	1 (2.1%)	8 (5.5%)	2 (3.6%)		1 (2.2%)	7 (4.5%)	3 (6.3%)		11 (4.4%)
Married	42 (89%)	132 (90%)	50 (89%)		41 (91%)	140 (90%)	43 (90%)		224 (90%)
Single	4 (8.5%)	6 (4.1%)	4 (7.1%)		3 (6.7%)	9 (5.8%)	2 (4.2%)		14 (5.6%)
Education				0.2				0.2	
	14 (30%)	58 (40%)	28 (50%)		15 (33%)	59 (38%)	26 (54%)		100 (40%)
>Secondary	9 (19%)	33 (23%)	12 (21%)		11 (24%)	37 (24%)	6 (13%)		54 (22%)
Secondary	24 (51%)	55 (38%)	16 (29%)		19 (42%)	60 (38%)	16 (33%)		95 (38%)
Income, n (JODs)				0.2				0.6	
<500	27 (57%)	64 (44%)	28 (50%)		24 (53%)	74 (47%)	21 (44%)		119 (48%)
500-1000	20 (43%)	82 (56%)	28 (50%)		21 (47%)	82 (53%)	27 (56%)		130 (52%)
BMI	31.8 (5.1)	31.6 (5.5)	30.7 (4.5)	0.5	31.9 (5.2)	31.2 (5.3)	31.8 (5.0)	0.5	31.4 (5.2)
DM duration	5.2 (2.9)	8.6 (5.3)	9.8 (4.9)	<0.001	6.9 (4.3)	6.7 (3.9)	14.3 (4.8)	<0.001	8.2 (5.1)
Glycemic control				0.024				<0.001	
Controlled	12 (26%)	19 (13%)	4 (7.1%)		17 (38%)	15 (9.6%)	3 (6.3%)		35 (14%)
Uncontrolled	35 (74%)	127 (87%)	52 (93%)		28 (62%)	141 (90%)	45 (94%)		214 (86%)
DM medications				0.2				0.4	
Insulin	1 (2.1%)	3 (2.1%)	3 (5.4%)		2 (4.4%)	5 (3.2%)	0 (0%)		7 (2.8%)
OHA	35 (74%)	99 (68%)	31 (55%)		31 (69%)	105 (67%)	29 (60%)		165 (66%)
OHA + Insulin	11 (23%)	44 (30%)	22 (39%)		12 (27%)	46 (29%)	19 (40%)		77 (31%)
Smoking status				0.3				0.4	
Former smoker	6 (13%)	10 (6.8%)	6 (11%)		6 (13%)	12 (7.7%)	4 (8.3%)		22 (8.8%)
Non-smoker	19 (40%)	81 (55%)	30 (54%)		24 (53%)	77 (49%)	29 (60%)		130 (52%)
Smoker	22 (47%)	55 (38%)	20 (36%)		15 (33%)	67 (43%)	15 (31%)		97 (39%)
HbA1c level	8.10 (1.64)	9.05 (1.88)	10.36 (2.03)	<0.001	7.83 (1.72)	9.20 (1.69)	10.28 (2.48)	<0.001	9.16 (2.01)
Comorbidities									
Dyslipidemia	24 (51%)	115 (79%)	42 (75%)	<0.001	23 (51%)	119 (76%)	39 (81%)	0.001	181 (73%)
Hypertension	19 (40%)	76 (52%)	27 (48%)	0.4	16 (36%)	75 (48%)	31 (65%)	0.019	122 (49%)
CVD	8 (17%)	32 (22%)	6 (11%)	0.2	6 (13%)	27 (17%)	13 (27%)	0.2	46 (18%)
Neuropathy	7 (15%)	30 (21%)	16 (29%)	0.2	8 (18%)	29 (19%)	16 (33%)	0.076	53 (21%)
Retinopathy	1 (2.1%)	11 (7.5%)	10 (18%)	0.017	4 (8.9%)	14 (9.0%)	4 (8.3%)	>0.9	22 (8.8%)
Nephropathy	2 (4.3%)	5 (3.4%)	5 (8.9%)	0.2	3 (6.7%)	8 (5.1%)	1 (2.1%)	0.6	12 (4.8%)

For the CPIS, a higher CPIS score (CPIS = 3-4) was associated with higher age (p-value < .001) and longer DM duration, and 45 (94%) patients with CPIS of 3-4 had uncontrolled DM (Table 3). Hb1Ac was significantly higher in patients with a CPIS of 3-4, 39 (81%) patients with a CPIS of 3-4 had dyslipidemia, and 31 (65%) had hypertension.

## Discussion

Periodontal diseases are caused as a result of a combination of factors, encompassing both individual-specific risks and insufficient oral care. Among individuals with DM, the presence of severe periodontal disease is associated with a higher risk of mortality when compared with those having no or mild periodontal disease [12]. Therefore, in this retrospective single-center study, we aimed to investigate the association between periodontal health and the presence of periodontal diseases in Jordanian patients with DM.

Our results demonstrated varying levels of plaque accumulation, gingival inflammation, papillary bleeding, and community periodontal status among diabetic individuals. A substantial proportion of patients exhibited intermediate to high plaque index scores, with a significant correlation with age, longer DM duration, and poor glycemic control. Plaque buildup and the incidence of gingivitis among diabetic adolescents and young adults were found to be higher compared to those in healthy counterparts [13]. Similarly, a study by Sadeghi et al. revealed that diabetic individuals had a higher plaque index in comparison to the control group, aligning with the findings of our study and other studies by Aren et al. [14,15]. However, Lopez et al. demonstrated no statistically significant difference in dental health status between the groups [16]. It is currently believed that chronic gram-negative periodontal infection exacerbates insulin resistance, thereby playing a role in the onset of metabolic imbalance. Additionally, the interaction between periodontal bacterial by-products and mononuclear phagocytic cells, along with fibroblasts, is recognized to stimulate the continual release of cytokines (IL-1β, IL-6, and TNF-α), PGE2, and CRP [17].

Gingival index scores, papillary bleeding index scores, and community periodontal index scores were also high, indicating compromised periodontal health in this population. A study by Apoorva et al. showed that patients with type 2 DM exhibited a significantly higher CPI score, indicating a higher prevalence of periodontal diseases among diabetic patients, and factors such as glycated hemoglobin (Hb1Ac), duration of diabetes, fasting blood sugar levels, personal habits, and oral hygiene practices demonstrated a positive association with periodontal damage [18]. Moreover, our findings showed that DM individuals with severe periodontal diseases were more likely to have comorbidities such as dyslipidemia, hypertension, and diabetic complications. Studies have shown that in some instances, individuals with DM might use calcium channel blocker medications like amlodipine and nifedipine to manage hypertension, potentially leading to gingival overgrowth. Additionally, medications may sometimes manifest other oral effects, such as lichenoid mucosal reactions associated with metformin [19,20]. In concordance with our results, a study by Kim et al. showed that the duration of diabetes, fasting blood glucose (FBG), and adherence to self-management of diabetes significantly affected periodontal indicators such as the count of missing teeth and papillary bleeding index. Additionally, the CPIS was notably impacted by the duration of diabetes, FBG levels, and HbA1c [21]. According to Kneckt et al., individuals who adhere well to diabetes self-management often exhibit greater dental self-efficacy, which correlates with improved periodontal health. Conversely, strong adherence to diabetes self-care may lead to better control of HbA1c levels, a factor linked to periodontal well-being [22]. A study conducted on 28,801 patients found that type 2 DM is more associated with periodontitis than type 1 DM, they have also found its association with poor glycemic control [23].

Our study provides several strong points. First, with 249 diabetic patients included, our study has a relatively large sample size, enhancing the robustness of the findings and allowing for more generalizable conclusions. Second, periodontal health was assessed using well-established indices like the plaque index, gingival index, papillary bleeding index, and community periodontal index, ensuring the reliability and validity of the measurements.

However, our study has some limitations. First, being a retrospective cohort study, the research is susceptible to biases inherent in retrospective analyses, such as selection bias and incomplete data. Second, conducting the study in a single diabetes clinic may limit the generalizability of the findings to other settings or populations. Conducting prospective studies with longer follow-up periods would allow for the assessment of temporal relationships between DM characteristics and periodontal health outcomes. Investigating the effectiveness of periodontal interventions, such as improved oral hygiene education or periodontal treatments, in improving glycemic control and reducing diabetes-related complications would be valuable. This cross-sectional study investigated the prevalence of periodontal disease and its association with diabetes characteristics among 249 type 2 diabetic patients in South Jordan. The study found a high prevalence of uncontrolled diabetes (86%), with significant comorbidities such as dyslipidemia (73%) and hypertension (49%). Periodontal indices revealed moderate to severe periodontal disease, with notable plaque accumulation, gingival inflammation, and bleeding upon probing. The findings underscored the interplay between poor glycemic control and increased periodontal disease severity. These results highlight the critical need for integrated diabetes and periodontal care strategies, suggesting that improved periodontal health could potentially enhance diabetes management. Further research is warranted to develop effective intervention strategies aimed at mitigating the bidirectional impact of diabetes and periodontal disease.

## Conclusions

Our study provides valuable insights into the association between DM characteristics and periodontal health in Jordanian patients. The findings highlight the importance of comprehensive oral health assessments and tailored interventions for diabetic individuals to mitigate the risk of periodontal diseases and associated complications considering the bidirectional relationship between diabetes and periodontal disease. Despite certain limitations, the study contributes to the growing body of evidence linking DM and periodontal health and highlights avenues for future research and clinical practice. Early detection and intervention strategies targeting periodontal health may contribute to better glycemic control and overall health outcomes in individuals with diabetes. Further research is warranted to explore the mechanistic links between DM and periodontal disease and to evaluate the efficacy of periodontal interventions in improving diabetes-related outcomes.
